# Climate for Consumptives

**Published:** 1872-04

**Authors:** 


### HALL'S

### JOURNAL OF HEALTH.

Vol. XIX. — APRIL, 1872. — No. IV.

### CLIMATE FOR CONSUMPTIVES.

If a man coughs more or less every day, but most on going to bed or getting up, without raising any phlegm, or very little, and has a pulse generally over eighty beats in a minute, he may be very safely pronounced to be in the first stages of consumption. Such a man will do best in a cool, dry, bracing atmosphere; not so cool but that he can be out of doors a great part of daylight without a feeling of chilliness.

If such a person goes to a warm climate, he will suffer injury, because warmth debilitates; it indisposes and unfits one for exercise, while exercise in the open air, for the greater part of daylight, is essential towards wearing out of the system the diseased, and decaying, and waste particles which clog its working, as also towards getting the largest supply possible of that pure, out-door air which is so essential in securing a new life to the blood.

The second stage of consumption, which is also the last, because it is the decaying stage, is when there is yellow matter coughed up every day, with troublesome shortness of breath on going along slight ascents, and a pulse nearly all the time in the neighborhood of a hundred every minute.

Such persons cannot get well. If it is warm, they are weakened, and have no ambition to leave the house; if cold, the out-door air chills them to the very heart, and they cannot face it. Hence, for persons in the first stages of consumption, out-door activity, in a cool atmosphere, for the greater part of daylight, is most promising of good.

If in the last stages, they should be where they could have out-door air without chilliness.

It is perfectly dreadful to have actual consumption, and to be a thousand miles away from home, and friends, and kindred, dependent for the smallest offices on the pity of friends, or on purchased attentions, so heartless and so hard; and yet it is safe to say that every year hundreds, if not thousands, are advised with flippant impertinence or inexcusable ignorance, "O, you ought to go to the South." "Minnesota is the place for you."

Medical men would do well to question their consciences closely, to ascertain if they do not advise consumptives now and then to make a change of climate because they want to get rid of them, knowing that nothing more can be done for them, and that their case is hopeless; and too often, without knowing whether they have the pecuniary ability to make the change; without knowing that it might not be financial ruin to adopt such a course. Such counsel is treacherous, is dishonorable, is inhuman, — to send a man away from home to die among strangers!

Crowds of consumptives have been sent to Minnesota, of late years, to recover their health; not merely those who are in the first stages, having only a slight daily cough, and a pulse about the eighties most of the time for months, but when large quantities of yellow matter are expectorated every day, with short breath, rapid pulse, bodily debility, and increasing thinness of flesh. This is doing them a great wrong, for official records show that for five months in the year the thermometer averages within sixteen degrees of zero in Minnesota, making it necessary to keep invalids in hot, stifling, close, stove rooms day after day and week after week, and for months together; how is it possible for them to get well! No wonder is it, that three fourths of all who go to Minnesota to be cured of consumption, die there — the exact statement being, "Of all who go there in the *early* stages of consumption, only one in fifteen is cured."

Our conviction, after a lifetime spent in the treatment of diseases of the lungs is, that as long as a man is able to attend to his business, so as to be out of doors in the open air, several hours at a time every day, he ought by all means, if he has consumptive symptoms, — cough, quick pulse, wasting of flesh, — to stay at home where he can command suitable food at suitable times, avoid hurtful or dangerous exposures, secure those attentions which only true friendship or affection can give; feel free to retire, to be quiet, to exercise when it is most agreeable; and instead of having the mind more or less discomposed all the time about home, and friends, and kindred far away, and business affairs, he can have it fully employed, and thus divert it from the incessant contemplation of the symptoms of disease, with all its injurious and depressing consequences.

If, on the other hand, a man with evident consumptive symptoms, those above named, is too weak to ride on horseback, he cannot recover anywhere else if he does not recover at home. Hence it is a cruelty to send him from home, wherever it may be, North, South, East, or West. Our meaning is, that if any man has consumption in its first or in its last stages, and he has a reasonably comfortable home of his own, his wisest and best plan is to stay there, or keep so near it that he can readily get there within a few hours.

Other things being equal, in any ordinary case of consumption, if a man has money enough, the chances of recovery from consumption are better in a large city than in the country, with all its boasted advantages of pure air, fresh vegetables, luscious fruits, spring chickens, rich butter, and fresh laid eggs; these things can be better obtained the year round, in their highest perfection, in New York city, than at the "farm-house." In addition, hot and cold water for all bathing processes are at hand in every dwelling, at any hour of the day or night; every room about the house is tidy, cosy, and comfortable. You can ride for miles, and for hours at a time, in an omnibus or a rail car for a few cents; or take a carriage drive in the Central Park every day, or make an excursion around the harbor, or out to sea, or up the Hudson. If muscular exercise is desired it can be had the year round; on the shady side of the street in warm weather, and on dry sidewalks in cold, with an infinite variety of attractions, and diversions, and changing panoramas in every five minutes of the day. And at night, instead of being cooped up in a warm room for all the weary hours of midwinter, from five to ten, or from sundown to bed-time, half the time in moody silence, or listless lounging, or doziness, there is the excitement of the lecture-room, the exhilaration of the concert, or the quieting agencies of the "weekly meeting," or the "house of prayer." For let it be remembered that when a man is in actual consumption his lungs are in a state of decay, his prominent symptom is shortness of breath, and no wonder, because part of the lungs are gone and a much larger portion is inactive, so that he can't get air enough; hence it follows that the air he does get should be the purest possible, and that of course is the out-door air, for there is none that is pure within any four walls under a roof.

The thing that is wanting in every case of consumption more than all others put together, is air, pure air; and whether at night or day it is better for the consumptive to be in it every hour of the twenty-four that is possible; the only one precaution needed at night is to keep in exercise sufficiently active to prevent a feeling of chilliness, for then a cold is impossible; while the exhilaration and exposure to out-door air in going to places of amusement at night, more than compensates for any trifling ill-effects of breathing the atmosphere of a crowded room for an hour or two.

It is true that the air in the country is purer than that of the city, but look at the obstacles to getting it in the country: the dusty roads in summer, the muddy roads in winter, the damp grass at morning and evening; the time, and trouble, and delay of a horseback ride, or in gearing up a vehicle; if it is a lady, she must have an attendant. On the other hand, in the city, it is only necessary to put on a hat and cloak at a moment's notice, and walk or ride at one's convenience.

No medicine known up to this time has ever had any appreciable effect in the direct cure of consumption, beyond what is better accomplished by —

1st. An abundant supply of out-door air.

2d. A good appetite.

3d. A vigorous digestion.

4th. Mind and body fully and pleasurably, profitably and absorbingly occupied.

5th. Keeping the bodily functions in healthful order.

All these can be better regulated and secured at home than elsewhere; hence the best place for a consumptive in all latitudes and localities, winter and summer, is at his own comfortable home.

